# Unraveling the Consequences of Oxygen Imbalance on Early Embryo Development: Exploring Mitigation Strategies

**DOI:** 10.3390/ani13132171

**Published:** 2023-07-01

**Authors:** Thamiris Vieira Marsico, Mara Viana Silva, Roniele Santana Valente, Kelly Annes, Vitor Braga Rissi, Werner Giehl Glanzner, Mateus José Sudano

**Affiliations:** 1 Center for Natural and Human Sciences, Federal University of ABC, Santo André 09210-580, SP, Brazil; thaamirisvieira@gmail.com (T.V.M.); marabiotec@gmail.com (M.V.S.); ronielevalente@gmail.com (R.S.V.); 2 Department of Genetics and Evolution, Federal University of São Carlos, São Carlos 13565-905, SP, Brazil; kellyannes@ufscar.br; 3 Faculty of Veterinary Medicine, Federal University of Santa Catarina, UFSC, Curitibanos 89520-000, SC, Brazil; vitor.rissi@ufsc.br; 4 Department of Animal Science, McGill University, Sainte-Anne-de-Bellevue, QC H9X 3V9, Canada; werner.glanzner@mcgill.ca

**Keywords:** oxidative stress, reactive oxygen species, in vitro production, embryo development

## Abstract

**Simple Summary:**

Early embryo development is a dynamic and orchestrated event. Oxygen plays a fundamental role in several key biological processes necessary to sustain and complete embryonic development. Variations in the atmospheric concentrations during preimplantation development can promote several modifications in metabolic function and, consequently, biological processes affecting embryonic competence. Here, we have reviewed the impact of oxygen tension on essential embryonic traits during early development and addressed strategies to mitigate harmful effects due to unbalanced oxygen tensions. Oxygen tension, therefore, is crucial for embryonic competence acquisition and pregnancy success of embryos produced in vitro.

**Abstract:**

Although well-established and adopted by commercial laboratories, the in vitro embryo production system still requires refinements to achieve its highest efficiency. Early embryonic development is a dynamic event, demanding suitable conditions to provide a high number of embryos with quality and competence. The first step to obtaining an optimized in vitro environment is to know the embryonic metabolism and energy request throughout the different stages of development. Oxygen plays a crucial role in several key biological processes necessary to sustain and complete embryonic development. Nonetheless, there is still controversy regarding the optimal in vitro atmospheric concentrations during culture. Herein, we discuss the impact of oxygen tension on the viability of in vitro-produced embryos during early development. The importance of oxygen tension is addressed as its roles regarding essential embryonic traits, including embryo production rates, embryonic cell viability, gene expression profile, epigenetic regulation, and post-cryopreservation survival. Finally, we highlight the damage caused by in vitro unbalanced oxygen tensions and strategies to mitigate the harmful effects.

## 1. Introduction

In vitro embryo production (IVEP) is a reproductive biotechnique with tremendous implications for the field of animal reproduction and has significant relevance for both commercial and research purposes. Factors such as temperature, pH, and high concentrations of oxygen (O_2_), can adversely influence the outcome of IVEP, impairing cell metabolism and decreasing embryonic development rates [1]. Despite being a well-known and widely used biotechnology in human and animal reproduction, IVEP has certain limitations, which can be assessed through morphological, metabolic, and genetic evaluations [2].

Studies focused on bovine IVEP demonstrate that comprehensive knowledge of cellular, molecular, and metabolic aspects is essential for a better understanding of early embryonic development [3]. It is well established that oxidative stress resulting from an accumulation of ROS is associated with high levels of O_2_, which can negatively impact the in vitro production of embryos [4]. Therefore, the O_2_ levels reaching the oocyte are fundamental for establishing meiotic competence and promoting embryonic development [5]. Indeed, using 20% O_2_ during the maturation of bovine oocytes significantly reduced viability when compared to the 5% O_2_ concentration [6]. Moreover, it is important to consider the effects of O_2_ tensions on in vitro embryo production at the beginning and the end of the production procedure. In the initial stage, embryos are exposed to 5% O_2_, and during development, the oxygen supply is reduced to 2%. Thus, these studies raise the question of whether the in vitro fertilization culture system should mimic this progressively hypoxic environment of in vivo production [7].

The in vivo O_2_ tension environment differs drastically from the atmospheric O_2_ tension. In the mammalian oviduct, the O_2_ tension is approximately 5–7%, whereas, in the atmospheric environment, it is around 20% [3,4]. Embryonic development in the oviduct relies on low O_2_ tension, which plays a crucial role in maintaining the balance between reactive oxygen species (ROS) production and antioxidant enzyme activities. This event occurs due to an efficient redox reaction system, which ensures an optimal environment for cell development [8]. In mammalian metabolism, the role of hypoxia-inducible factor (HIF) as a crucial transcription factor for cell survival under low O_2_ conditions has been extensively documented in the literature. In this study, we have chosen to focus on areas other than the well-established HIF1-alpha-dependent effects of oxygen tension as it is well-reviewed elsewhere [9,10,11].

One of the biggest problems associated with higher O_2_ tension culture systems is the accumulation of ROS, which are formed as a result of O_2_ reduction reactions, leading to elevated free radicals. Free radicals are a physiologic consequence of cell metabolism and physiology, but in excess, they can cause irreversible damage [3]. Embryo development in mammals such as mice, hamsters, rabbits, pigs, and sheep can be affected by the accumulation of ROS, resulting in oxidative stress and the formation of hydrogen peroxide, which induces DNA damage in cells under in vitro conditions [12]. Nonetheless, ROS alters various types of cellular molecules, including DNA, proteins, lipids, as well as membranes, mitochondria, and endoplasmic reticulum structures, and it also induces developmental blockage and delays [13]. Therefore, this review aims to highlight how different O_2_ tensions can impact the viability and quality of in vitro-produced (IVP) embryos and to provide evidence about the importance of oxygen in the mechanisms involved in cell metabolism while also pointing out strategies used to mitigate the negative effects of O_2_ on embryonic cell culture.

### 1.1. Importance of O_2_ Tension for In Vivo and In Vitro Embryo Production Systems

Oxygen is crucial for metabolic balance during embryonic development, as it is indispensable for releasing chemical energy and providing nutrients to the cells. Thus, the local oxygen tension plays a pivotal role in affecting physiological processes [14]. Embryonic oxidation and other forms of energy production rely on the continuous supply of oxygen to sustain embryonic development and regulate stem cell fate, morphogenesis, and organogenesis [13]. Therefore, the control of the gaseous atmosphere becomes a stress factor during embryo culture, as excessive oxygen levels combined with ammonium can disrupt the glutamine and alanine transamination pathway, thereby negatively impacting fetal development [15].

Both intrinsic and extrinsic factors, including ions, buffers, growth factors, amino acids, energy substrates, and the gaseous atmosphere, can influence early in vitro embryo development [6]. Culturing embryos under atmospheric O_2_ tensions in vitro has been observed to result in increased ROS production, which affects embryonic growth [3,16,17,18]. Additionally, different O_2_ tensions have been shown to alter embryo transcriptome, proteome, carbohydrate, and amino acid metabolism, embryo homeostasis, and epigenome. This includes the induction of premature X chromosome inactivation, with potential differential effects on male and female embryos [15,19,20,21,22]. In contrast, embryos in the in vivo environment experience physiological concentrations of oxygen, and studies suggest that the uterine oxygen tension may be lower than that in the oviduct [23]. This suggests that culture embryos under 2% O_2_ or lower from the morula to the blastocyst stage can promote the development of healthier embryos [24].

One of the main distinctions between in vitro and in vivo embryo production lies in the oxygen concentration in the environment, highlighting the need to closely monitor and control O_2_ levels in in vitro conditions. Changes in O_2_ levels within the in vitro environment can affect physiological processes during embryo production. During the early embryonic stage, physiological hypoxia is crucial for the development of organs and tissues, such as the heart and cartilage, as lower oxygen levels stimulate the migration of multipotent cells [25]. Elevated O_2_ levels can modulate the activity of the insulin-like growth factors (IGF) system, which is one of the signaling pathways responsible for regulating embryonic growth rates [26]. These changes significantly impact cell metabolism, gene transcription, chromatin, and nucleosome arrangements [27].

In in vitro embryo production, environmental conditions are simulated to create an artificial system capable of maintaining and sustaining embryo development with a growth potential similar to embryos developing in vivo. However, fluctuations in O_2_ tension can affect crucial stages of the production process, such as the buffering of the culture medium [28]. The buffering of the media depends on the gaseous composition used during embryo culture and can also be influenced by manipulations performed outside the incubator [29].

### 1.2. Effects of Oxygen Concentration on Embryonic Cell Viability in the IVEP System

The IVEP system encompasses various procedures aimed at supporting normal embryo growth and development while mimicking a physiological environment. Oxygen, among other factors, plays a critical role in embryonic cell respiration and energy metabolism [30]. The difference in O_2_ tension between in vivo and in vitro environments is particularly important. The variation in O_2_ concentrations, specifically between physiologic levels (~5%) and atmospheric levels (20%), directly impacts the overall metabolic status of preimplantation embryos in the culture system [6]. However, the exact mechanisms underlying the detrimental effects of higher O_2_ tension on embryonic competence and quality are not yet fully understood.

One widely accepted aspect recognized by the scientific community is the accumulation of ROS, leading to increased oxidative stress (OS). During the oxidative phosphorylation pathway, oxygen is consumed, and high-energy electrons generate ROS (commonly known as free radicals) [31]. Free radicals are chemical species (atoms, molecules, or ions) with one or more unpaired electrons in their outer shell, formed through redox reactions by the rupture of a chemical bond. Examples include hydroxyl (OH·), nitric oxide (NO·), superoxide (O_2_·−), peroxyl (ROO·), nitrogen dioxide (NO_2_·), and lipid peroxyl (LOO·). Additionally, oxidants such as lipid peroxide (LOOH), nitrous acid (HNO_2_), singlet oxygen (1O_2_), hydrogen peroxide (H_2_O_2_), ozone (O_3_), hypochlorous acid (HOCl), peroxynitrite (ONOO–), and dinitrogen trioxide (N_2_O_3_) can trigger radical reactions [32].

**Figure 1:**
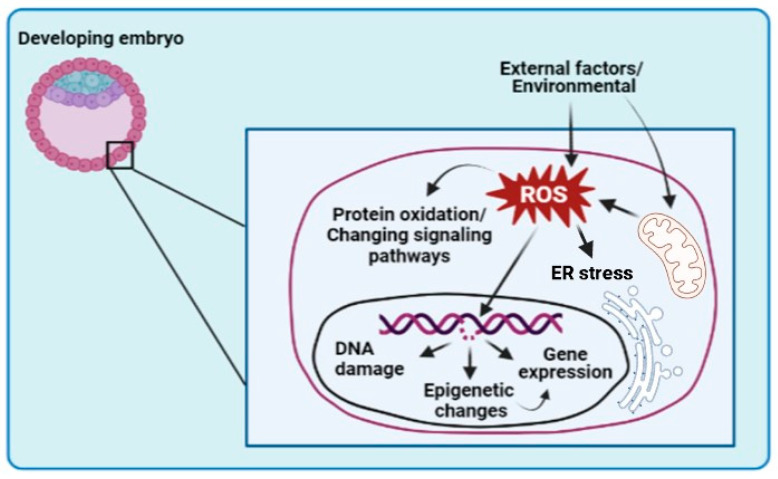
Effect of reactive oxygen species (ROS) on embryonic cells. Environmental factors and mitochondria are sources of ROS generation. Excessive ROS causes changes in cellular DNA, endoplasmic reticulum stress, epigenetic profile, and protein and lipid degradation, amongst other problems, compromising embryonic development.

Due to their unstable nature and availability of an electron for reaction, free radicals typically result in DNA damage and compromised production of proteins and lipids—critical molecules responsible for cell membrane stability and overall cellular function and signaling [3] (Figure 1). Studies on porcine embryos have demonstrated reduced H_2_O_2_ production and DNA fragmentation at 5% of the O_2_ atmosphere compared to 20% [12]. Mouse embryos, under low O_2_ tension, prioritize the oxidative utilization of pyruvate [33], while sheep embryos show increased catabolic utilization of glucose [34]. Currently, to improve embryo quality at the cleavage and blastocyst stages, it is recommended to culture embryos under ultra-low concentrations (2%) to mitigate oxidative damage. Conversely, supraphysiological oxygen levels (20%) have been shown to be toxic, while reduced O_2_ tension (5–7%) during embryo culture has been extensively documented to have advantages in various mammalian species, including hamsters [4,28], rats [35], pigs [36,37], mouse [6,29], goat [38], humans [1,39], sheep [40,41], and cow [40,42].

Oxygen enters the cell through passive diffusion, regulated by various physicochemical factors. Oxygen consumption during oxidative phosphorylation is influenced by substrate availability and enzyme complexes in the inner mitochondrial membrane. Oocytes possess approximately 100 times more mitochondria than somatic cells [43], making mitochondrial integrity crucial. When the mitochondrial respiratory chain is compromised, free radicals are produced [44]. As the embryo grows and differentiates, the mitochondrial respiratory chain becomes the primary source of essential energy while also being a major contributor to the production of ROS [45].

Mitochondrial DNA (mtDNA) is particularly vulnerable to damage as it lacks protective proteins such as histones and repair systems. Consequently, mtDNA mutation rates increase, leading to harm in transcripts and genomic DNA. These mutations impair protein synthesis, including enzymes involved in the oxidative phosphorylation chain, resulting in further production of free radicals due to potential electron loss [3]. Mitochondrial injuries, caspase activation, and apoptosis have been associated with lower developmental competence in preimplantation embryos [46]. Moreover, increased ROS levels have been observed in the IVEP of various species, such as cattle [47], mice [48], and humans [29].

Preimplantation embryos are highly susceptible to ROS, which are produced by various enzymes and metabolic pathways, negatively affecting their production, quality, and development [49,50]. Embryos undergo a crucial process of replacing maternal RNA with embryonic RNA, necessitating new protein synthesis [51]. The endoplasmic reticulum (ER) plays a significant role in the biosynthesis of proteins, lipids, and secretory proteins, serving as the major intracellular compartment responsible for protein folding and processing. Proper protein folding in the ER is essential for the secretion of functional proteins [52,53].

ER quality control (ERQC) ensures the proper protein folding, where correctly folded proteins are exported to the Golgi complex, while misfolded proteins are either given a chance to refold correctly or targeted by chaperones for degradation through ER-associated degradation (ERAD) [54]. The response to ER stress is known as the unfolded protein response (UPR), which maintains cellular homeostasis. However, if the accumulation of misfolded proteins exceeds the ER’s capacity, apoptotic signaling may be initiated [55].

Gametes and developing embryos in in vitro culture systems can be subjected to various types of exogenous stress [56]. Adverse factors are known to negatively impact ER functions and protein synthesis, leading to the activation of ER stress and the UPR signaling pathways. For example, the treatment of embryos with *Tunicamicyn*, an agent that induces protein glycosylation and subsequent ER stress during in vitro culture, impairs embryo development by reducing blastocyst formation and cell number and inducing apoptosis in many species [57]. Similarly, embryos cultured under 20% O_2_ exhibit the upregulation of ER stress markers and increased OS [58].

The activation of ER stress pathway triggers the UPR, which aims to restore cellular homeostasis. However, if the factors causing ER stress are severe or prolonged and homeostasis is not reestablished, apoptosis may occur [59]. The UPR signaling pathway involves three ER transmembrane proteins: PERK (double-stranded activated protein kinase-like, ATF6 (activated transcription factor 6), and IRE1 (inositol-requiring enzyme 1), along with the ER molecular chaperone BiP (immunoglobulin-binding protein, also known as glucose-regulated protein 78, or GRP78). Under normal physiological conditions, BiP interacts directly with PERK, ATF6, and IRE1, which are transmembrane proteins of the ER. However, when unfolded or misfolded proteins accumulate, BiP is released from these transmembrane proteins, triggering UPR activation [60].

The PERK signaling pathway decreases protein translocation into the ER lumen to prevent protein accumulation, while the ATF6 and IRE1 pathways modulate the transcriptional activation of genes responsible for increasing translocation, protein folding, export, degradation, and other ER functions [61]. The release of IRE1 from BiP induces the splicing of Xbp1 (X-box binding protein-1). The spliced form of Xbp1 (sXbp1) encodes a transcription factor that promotes the transcription of ER chaperones, thereby maintaining cellular homeostasis through its involvement in ER protein folding. XBP1 plays a critical role in porcine oocyte maturation, early embryo development, and embryonic genome activation [62].

Increased ER stress during preimplantation embryo development triggers the UPR, negatively affecting blastocyst formation [63]. ROS enhances the expression of ATF4 (activating transcription factor 4) and CHOP (DNA damage-inducible transcript 3, also known as C/EBP homologous protein/CHOP), which are genes induced by UPR activation and negatively affect bovine embryos development. Increased ROS levels also upregulate ATF6 expression, leading to an increased expression of BiP, Xpb1, and CHOP, resulting in decreased blastocyst formation [64]. It has been reported that when the O_2_ concentration is similar to in vivo conditions, the chances of ER stress and UPR activation are minimal, but the competence of bovine embryos depends on the coupled response between oxidative stress and ER stress [58].

ROS can be produced in all cellular compartments and ultimately results in protein damage [65]. There is growing evidence suggesting that protein folding and the generation of ROS as products of protein oxidation in the ER are closely related events. Oxidative protein folding occurs in the ER, and disruptions in protein folding may lead to alterations in redox status or the generation of ROS, directly and/or indirectly affecting ER homeostasis and protein folding [66]. Further research is needed to fully understand these processes in preimplantation embryos.

In addition to protein damage, lipids can be affected by the oxidation of cellular membranes by hydroxyl free radicals, resulting in modifications of physicochemical properties, such as eutectic points [3]. ROS also target cytoplasm compounds, disrupting the ratio between glutathione and glutathione disulfide and increasing intracellular calcium ion concentration, which initiates pathological events [67]. Interestingly, previous studies have shown the impact of O_2_ tension variations and increased OS on exosome (EXO) release and content [8,68]. Exosomes are carriers of transcripts, proteins, microRNAs, growth factors, and cytokines, and they are considered major mediators of cell-to-cell communication during in vitro bovine embryo production [69,70].

Overall, oxygen concentration plays a critical role in embryonic cell viability and development in the IVEP system. Deviations from physiological oxygen tension can lead to increased oxidative stress, ROS production, DNA damage, compromised protein and lipid synthesis, mitochondrial dysfunction, ER stress, and activation of the unfolded protein response. These factors collectively contribute to impaired embryonic competence and quality. Researchers have suggested that maintaining ultra-low oxygen concentrations (e.g., 2%) during embryo culture may help mitigate the detrimental effects of oxidative stress. However, further studies are needed to elucidate the precise mechanisms involved and to optimize the oxygen conditions for successful embryonic development in the IVEP system.

### 1.3. Oxygen Tension Affects Gene Expression Profile, Oocyte Competence, Development Rates, and Post-Cryopreservation Survival of In Vitro-Produced Embryos

It is known that the in vitro environment induces changes in cell metabolism, especially during preimplantation embryo development [71]. In recent years, the significance of O_2_ tension has been highlighted as a key factor affecting early embryonic development. While the O_2_ concentration in vivo is lower than the atmospheric air [72], for a long time, the IVEP utilized atmospheric O_2_ tensions of approximately 20%, evaluating embryo viability and competence based on morphology and blastocyst rates [73]. However, with technological advancements, biochemical and molecular analyses have revealed disorders in embryonic development that may not be apparent morphologically. 

Considering the dynamic nature of the oviduct and uterine environments and their close connection to in vivo embryonic development, the ideal O_2_ concentration for the IVEP remains a topic of debate. Nevertheless, numerous studies have adopted 5% O_2_ as the optimum level, primarily due to the O_2_ tension in the mammalian oviduct, which is around 5 to 7% [4]. Research indicates that embryos cultured in 5% O_2_ exhibit a higher blastocyst production rate, and greater total cell numbers, particularly in the inner cell mass (ICM) and trophectoderm (TE) [4,74,75]. Similarly, in ovine species, low O_2_ tension during in vitro fertilization (IVF) accelerates blastocyst formation kinetics [47]. These findings suggest that lower O_2_ tensions support embryonic development, promoting increased cell numbers. 

Moreover, studies on embryo cryopreservation have reported that embryos cultured at lower O_2_ tension display an increased hatching rate after warming [76,77]. Biochemical analysis has shown that embryos cultured in 20% O_2_ exhibit higher accumulation of ROS, increased mitochondrial abnormalities, lower mtDNA copy number, and decreased mitochondrial membrane potential [28,78]. Furthermore, O_2_ tension influences gene transcriptional profiles, as embryos cultured at 20% O_2_ demonstrate higher expression of genes related to apoptosis, oxidative stress response, antioxidant response elements, and mitochondrial gene expression changes [4,28,78]. Conversely, embryos cultured at 5% O_2_ show elevated expression of genes associated with embryonic development, cell proliferation, and cell fate, as well as higher DNA methylation at the 4-cell and blastocyst stages and lower repressive and permissive epigenetic marks [28,79,80]. Furthermore, global gene expression patterns indicate that mouse embryos cultured at 5% O_2_ align more closely with in vivo-derived (IVD) embryos [42]. The differently expressed genes (DEG) and differentially expressed proteins (DEP) in bovine and buffalo embryos exposed to 5% and 20% oxygen, respectively, are summarized in Table 1 [28,76]. Additionally, DEG and metabolites in in vitro and IVD mouse embryos are presented in Table 2 [42,81,82].

Interestingly, while mouse embryos cultured at 20% O_2_ may appear morphologically normal, they exhibit lower pregnancy rates and reduced development into fetuses [75,80]. In human embryos, pregnancy and live birth rates do not consistently improve when cultured at 5% O_2_; however, enhanced embryonic competence has been observed [80,85].

Metabolic profiling has emerged as a valuable tool for understanding cellular processes, allowing non-invasive analysis of spent culture media used for embryo culture and development. While it represents an alternative to avoid compromising embryo viability, culture media may obscure vital information due to metabolite degradation throughout culture, particularly when embryos are cultured in groups. For instance, a study analyzing spent culture media from human cleavage-stage embryos under 6% and 20% O_2_ found no significant changes in the depletion or release of relevant metabolites [84]. Thus, it would be premature to conclude that there are no differences between both groups of embryos solely based on the metabolic profile derived from spent culture media.

**Figure 2:**
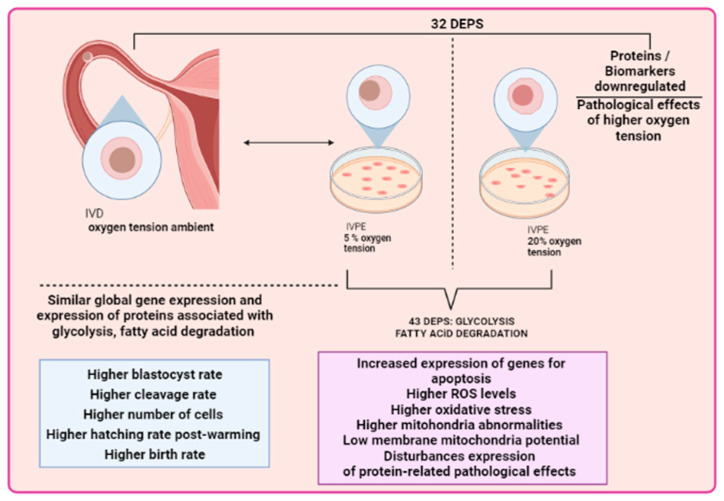
Comparison between 5% and 20% oxygen tensions between in vitro and in vivo embryos. Physiological O_2_ (5%) resulted in increased blastocyst yield, hatching rate, and cell count compared to atmospheric O_2_ (20%). Embryos produced in 5% O_2_ had improved post-cryopreservation survival. Proteome comparison of hatched blastocysts cultured at 5% vs. 20 % of O_2_ identified 43 differentially expressed proteins (DEPs) associated with crucial biological processes. Other variables such as birth rate, blastocyst rate, cryosurvival, and regulatory protein expression were considerably great in embryos cultured under 5% O_2_ and in vivo-derived embryos. Embryos exposed to 20% oxygen tension had these rates reduced and higher expression of negative regulatory proteins, which could lead to pathological effects.

Notably, proteomic profiles of embryos cultured at 5% and 20% O_2_ reveal distinct patterns between these two groups, highlighting differentially expressed proteins associated with metabolic processes such as glycolysis and fatty acid degradation [22,76] (Figure 2). Furthermore, embryos cultured at 5% O_2_ display morphological similarity to IVD embryos [19]. In line with this, bovine embryos cultured in vitro exhibit increased glucose metabolism compared to IVD embryos [83]. Additionally, embryos cultured at 5% O_2_ demonstrate reduced lactate production from glucose metabolism when compared to those cultured at atmospheric O_2_ tension [83,86]. However, a recent study on buffalo embryos attributed the observed metabolic profile under low oxygen tension to the Warburg Effect, which was characterized by less efficient aerobic glycolysis, increased glucose uptake, and lactated production even in the presence of O_2_. The authors suggest that this effect, along with modifications in lipid metabolism, improves in vitro embryo competence [76]. This metabolic strategy supports the rapid cell proliferation seen in preimplantation embryos [13,77]. The major results of these articles [22,76] are highlighted in Figure 2.

The increased expression of proteins from the SCL2A3 family, responsible for glucose uptake, observed in embryos cultured at low O_2_ tension, is suggested as a marker of embryos with high competence. Furthermore, elevated levels of glycolytic intermediates are associated with nucleic acid production and the maintenance of the cellular redox status [76].

Taken together, these findings underscore the importance of O_2_ tension as a critical variable affecting embryo quality, competence, embryo transfer, and success after cryopreservation. Atmospheric O_2_ tension is increasingly being replaced by lower levels, as low O_2_ concentration promotes improved embryo development by reducing ROS levels, while in vitro culture under 20% O_2_ affects gene expression and protein synthesis. These metabolic disturbances influence various commercial applications, such as cryopreservation, through the activation of signaling pathways related to apoptosis, lipids metabolism, and energetic dysregulation.

### 1.4. Epigenetic Effects of Oxygen Tensions during Embryo Development

It is widely documented that the regulation of epigenetics and chromatin configuration plays a crucial role during early embryo development [87]. This includes embryo genome activation, cell lineage specification, and ultimately blastocyst formation in various mammalian species such as mice [88,89], bovine [90,91], and porcine [91,92,93], among others. To ensure proper epigenetic regulation, the energetic metabolism must provide the necessary energy substrate, as most of the epigenetic factors (writers and erasers) and epigenetic modulations are closely linked to cell metabolism in early embryo development [94,95]. Oxygen tension also plays a role in the epigenome during embryo development [96]. This is evidenced by modifications in DNA methylation patterns in response to oxidative stress in cells [97]. Additionally, in embryonic stem cells, O_2_ concentration modulates important epigenetic regulators in terms of DNA methylation and histone modifications, which in turn significantly impacts imprinting regulation and activation of pluripotent genes, thereby affecting embryo viability and competence [98].

As previously mentioned, oxygen tension has significant implications on cell metabolism and, consequently, embryo development. Studies have demonstrated that different oxygen tension (5% versus 20%) during bovine oocyte in vitro maturation can affect cumulus expansion and lead to different levels of global DNA methylation in parental nuclei after fertilization, particularly in paternal nuclei [99]. Similarly, high oxygen tension (20% O_2_) in embryos has been shown to increase global DNA methylation patterns in bovine 4-cell stage embryos and modulate the expression of DNMT3a at the same stage of development without disrupting the expression of analyzed retrotransposons [21]. This is crucial as retrotransposons and transposable elements are known to be involved in embryo genome activation, acquisition of transcriptional profiles, and preimplantation development [100]. In bovine embryos, oxygen tensions have also been reported to alter histone marks such as repressive marks (H3K9me2) and permissive marks (H3K4me2) in blastocysts cultured in 5% versus 20% oxygen, thereby affecting chromatin configuration [79]. Interestingly, these studies did not observe any differential expression of the main regulators of these specific marks in both the morula and blastocyst stages [79]. Conversely, when mouse placental tissues were analyzed for DNA methylation patterns, no significant difference was observed in placenta-derived from embryos cultured in 5% or 20% oxygen tension, although abnormalities were observed when comparing the in vitro system to natural conception [101].

Although some evidence suggests that the different O_2_ tension affects epigenetic marks and their regulation throughout embryo development, further studies are needed to comprehend the exact mechanisms by which oxygen influences epigenetic programming and the consequences for in vitro embryo production systems.

### 1.5. Strategies to Mitigate the Harmful Effects Caused by Oxygen during In Vitro Embryo Production

In recent decades, there has been a significant increase in the number of IVP embryos in both human and animal species. With the expansion of this biotechnology, there has been a growing focus on understanding embryonic metabolism and cell survival mechanisms. This increased knowledge has shed light on numerous signaling pathways involved in embryonic development and competence. Consequently, new protocols and parameters are emerging every day to improve production results and embryo quality. However, the in vitro environment often falls short, providing reproductive cells with all the necessary nutrients and substances for successful fertilization, blastomeres differentiation, and early embryonic development.

In comparison to IVD embryos, IVP embryos still exhibit various structural, cellular, and metabolic differences. Thus, efforts have been dedicated to providing IVP embryos with an environment that closely resembles their physiological in vivo counterparts. One aspect that has received considerable attention is the role of O_2_ tensions during in vitro culture. Fluctuations in O_2_ concentrations have been found to be potentially harmful, leading to investigations into the detrimental effects of different O_2_ levels during this crucial period.

One strategy currently employed is the reduction of the O_2_ concentration, particularly during the embryo development period. Traditionally, the O_2_ concentrations of either 5% (physiological) or 20% (atmospheric) have been used in IVF and culture, with some variation throughout cultivation. However, many laboratories are now opting for complete cultivation at low tension. Initially, most embryology clinics conducted their procedures under atmospheric O_2_ conditions, primarily due to cost considerations associated with reducing the O_2_ concentration in the culture environment [30].

Several studies have investigated the effects of high (20%) and low (5%) O_2_ concentrations at specific stages of IVEP. For instance, a study using a mouse model examined the influence of these two O_2_ concentrations during the in vitro oocyte maturation (IVM) period [102]. Although there was no difference in blastocyst formation rates, embryos matured under low O_2_ tension exhibited a greater number of cells, indicating improved embryo quality and cryotolerance [103]. Similarly, a study involving bovine embryos found that the oocytes matured under 5% O_2_ resulted in a higher rate of blastocysts development compared to those matured under 20%. Moreover, oocytes matured under low tension showed lower intracellular H_2_O_2_ levels compared to those matured under higher tension [6].

Following fertilization, the presumptive zygotes undergo in vitro culture (IVC) for approximately 6 to 8 days until the formation of blastocysts. As the longest step in the IVEP process, the effects of O_2_ levels during this phase are more pronounced. In a factorial study focused on porcine embryo development, the effect of two O_2_ concentrations (5 and 20%) during both IVM and IVC was investigated [104]. The study found no difference in oocyte maturation rates, but the blastocyst rate was significantly higher in the 5% IVC group compared to the 20% IVC group. Interestingly, the group in which oocytes were matured at 20% O_2_ and cultivated at 5% O_2_ showed even greater blastocyst development indicating the potential for variations throughout the culture. Conversely, a study involving bovine embryos cultured under 5% or 20% O_2_ during IVC found similar blastocyst rates and the total number of cells at D7 [105]. It is worth noting that in this study, the embryos produced under higher O_2_ tension were co-cultured with cumulus cells, which might have attenuated OS.

**Figure 3:**
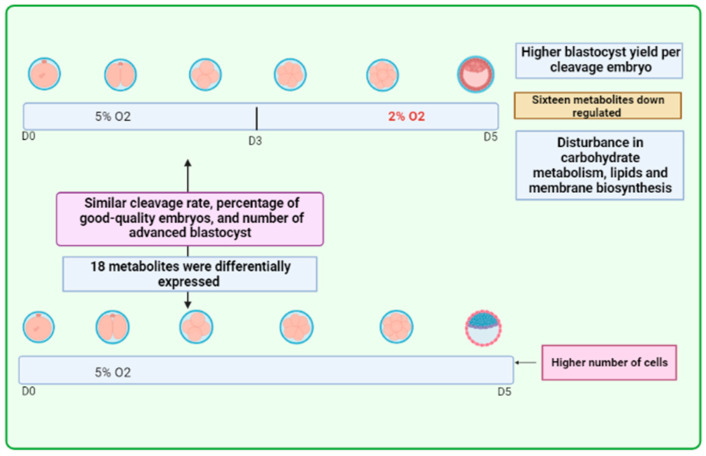
Embryos were cultured in 5% oxygen (O_2_) from day 1 (D1) to D3 and then to 2% O_2_ (from D3 to D5). This resulted in improved blastocyst yield when compared to constant exposure to 5% of O_2_. This system altered the gene expression and the main metabolic processes, including the metabolism of carbohydrates, lipids, and membrane biosynthesis. In constant exposure to 5% O_2_, the number of cells was more satisfactory than in reduced exposure to 2%.

More recently, studies have proposed the use of ultra-low O_2_ tension (2%) in embryo production. Data collected from different species of mammals have demonstrated that the O_2_ tension in the uterine environment is lower than in the oviduct region, typically around 2% [72]. In an attempt to mimic the embryo’s natural environment from fertilization to implantation in the uterus, a study with human embryos proposed sequential exposure to 5% O_2_ from days 1 to 3, followed by 2% O_2_ from days 3 to 5, as compared to continuous exposure to 5% O_2_ [7]. Embryos subjected to reduced O_2_ showed higher rates of high-quality blastocysts. However, limited information is currently available regarding this new approach, and more data is needed to reach a consensus. The main findings of this article are summarized in Figure 3.

The exact mechanism underlying the improved development of embryos cultured under low O_2_ is not yet fully understood, but mitochondria are likely to play a key role in mediating this effect [78]. Mitochondria are the primary generator of ROS, with 0.2–2% of the O_2_ taken up by the cells converted to ROS by mitochondria [106]. An imbalance between ROS production and antioxidant molecules can lead to various types of injury in embryonic cells, resulting in reduced cell viability (Figure 1). To counteract the potential damage caused by excessive ROS production, supplementing culture media with antioxidant compounds is a widely used alternative.

One important antioxidant synthesized in cells is glutathione (GSH). GSH plays a critical role in protecting cells from oxidative damage and maintaining redox homeostasis [107]. The supplementation of bovine IVM medium with glutathione ethyl ester (GSH-OEt) has been shown to preserve mitochondrial distribution patterns, decrease both cytoplasmic and mitochondrial ROS contents, and regulate the expression of genes involved in the OS [108]. Similarly, porcine oocytes treated with resveratrol during IVM exhibited increased intracellular GSH levels, reduced ROS levels, higher blastocyst formation rates, and a greater total cell number [109]. Other molecules, such as FLI (FGF2, LIF, and IGF2) in combination with melatonin and cysteine, have also shown improved porcine IVF parameters fertilization rates and higher cleavage and blastocyst rates under atmospheric O2 tension [110]. Combinations of two or more antioxidants have yielded satisfactory results highlighting their potential as alternative approaches. A list of studies conducted using antioxidants and their main results is described in Table 3 [111].

In addition to antioxidant supplementation, alleviating RE stress has been employed as a valid strategy to reduce apoptosis and enhance oocyte maturation and embryo development. *Tauroursodeoxycholic acid* (TUDCA), a bile acid that acts as a potent chemical chaperone to inhibit ER stress in vitro, has shown beneficial effects by suppressing the UPR [112]. Studies involving TUDCA supplementation during IVC have demonstrated improved embryo development and increased implantation and live birth rates in mice [113], reduced ER stress and ROS levels, improved embryo development [58], and enhanced cryotolerance [114]. Furthermore, TUDCA treatment has been found to enhance DNA damage repair and promote porcine preimplantation development [115]. These findings indicate that ER stress and associated developmental damages, exacerbated by high O_2_ tension and/or other factors, can be mitigated through the reduction of O_2_ tension and the use of antioxidants or ER stress inhibitors. Another commonly employed alternative is the use of buffered media when handling cells outside the incubator for extended periods, such as during intracytoplasmic sperm injection (ICSI) and vitrification. To protect embryos from harmful effects of prolonged exposure to atmospheric conditions, especially varying O_2_ concentrations, specific media can be used. The most common options include HEPES-buffered media, phosphate-buffered saline, and regular bicarbonate-buffered culture media [24].

Among all the available strategies, the most suitable approach depends on the specific production system, taking into consideration factors such as atmospheric conditions, cell type, cultivation protocol and duration, and other variables that may influence each process.

**Table 3:** Studies with the addition of antioxidant compounds to the embryonic culture medium and main results.

Component	Species	Reference	Results
Resveratrol	Porcine	(Kwak et al., 2012) [109]	Higher blastocyst formation rates, higher total cell numbers, decrease in ROS levels.
Melatonin	Mouse	(Gao et al., 2012) [116]	Suppressed ROS production and promoted embryonic development in vitrified mouse embryos.
Acetyl-l-carnitine, *N*-acetyl-l-cysteine, and α-lipoic acid	Mouse	(Truong et al., 2016) [111]	Increased the blastocyst cell number, maintained intracellular glutathione (GSH) levels, and improved fetal development irrespective of incubator oxygen concentration.
B-mercaptoethanol	Buffalo	(Moussa et al., 2019) [117]	Improved the quality of vitrified blastocyst evidenced by the modulation of the expression of blastocyst important genes, β-catenin, E-cadherin, and Oct-4, and the ability to protect vitrified blastocyst against apoptosis.
Ascorbic acid	Porcine	(Martín-Romero et al., 2008) [118]	ROS levels and survival rates after vitrification-warming were significantly improved. Addition of AC into vitrification-warming media enhanced embryo survival and embryo quality after warming.
Ascorbic acid	Porcine	(Nohalez et al., 2018) [119]	Increased the survival of in vitro-produced porcine blastocysts by decreasing ROS production.
Glutathione Ethyl Ester	Bovine	(García-Martínez et al., 2020) [108]	Before vitrification, supplementation of IVM medium with GSH-OEt preserved mitochondrial distribution patterns and diminished both cytoplasmic and mitochondrial ROS contents.
Glutathione and cysteine	Porcine	(Li et al., 2014) [120]	GSH or cysteine can improve the developmental competence of porcine ICSI-derived embryos by reducing intracellular ROS levels and the apoptosis index.
Lycopene	Bovine	(Chowdhury et al., 2018) [121]	There was a significant increase in cleavage and blastocyst development rates and a reduction of intracellular ROS concentrations in oocytes and blastocysts.

## 2. Conclusions

To enhance the chances of pregnancy success following embryo transfer, it is vital to establish favorable conditions that support the intricate cellular and molecular processes during early embryo development. In addition to formulating an appropriate culture medium, the oxygen levels to which embryos are exposed play a critical role in determining the outcome. Oxygen is essential for metabolic functions and various biological processes. However, when there is an imbalance between oxidative agents and antioxidant defenses, excessive oxygen can become detrimental to cells, leading to oxidative stress. This OS can result in DNA damage, disruptions in cell signaling, and alterations in protein expression—key factors that influence embryo development, quality, and pregnancy success.

To address this issue, several approaches have been proposed to minimize the potential harm caused by oxygen exposure. One such approach involves culturing embryos under low oxygen tension, which has demonstrated positive effects such as improved blastocyst rates, increased cell number, and molecular expression patterns closely resembling those observed in in vivo-derived embryos. Furthermore, the introduction of antioxidants has shown promise in mitigating the damage caused by disrupted in vitro culture systems. These advancements in biotechnology, coupled with the initial findings, pave the way for enhancing IVEP through optimized and sustainable cultured systems.

## Figures and Tables

**Table 1:** Differentially Expressed Proteins (DEP) in buffalo embryos and Differentially Expressed Genes (DEG) in bovine embryos cultured at 5% and 20% oxygen tension. The table shows upregulated proteins or genes in each species [28,76].

	Upregulated Proteins in Each Group	
Embryonic Species	5% O_2_	20% O_2_
Buffalo	DRG1, HMGCR, ISYNA, NSDHL, ASNS, MSH2, SCL2A3, SAS, PRXL2A, ACSL4, ACAT2, GAPDH, ACLY, ENO1, LDHA, COPA, PKM, TPI1, HMGCS1, PGK1	PTI, MVP, ITGB1, ILF2, GNS, ATP5IF1, KRT10, KRT6B, KRT2, HSPH1, TRIM23, ATP6V1E1, KRT1, C15H11orf58, ANXA1, BASP1
Bovine	ATF4, CDX2, DDIT3, KEAP1, OTX2, HSF1, PAF1, POU5F1, REST, SREBF1, XBP1	HAND1, NANOG, NFKB2, SOD2, SOX2

**Table 2:** Differentially expressed genes (DEG) in in vitro-produced mouse embryos compared to in vivo-derived mouse embryos. Metabolites of in vitro-produced mouse embryos [45,83,84].

**Microarray (22.000 Transcripts)**	
**5% O_2_ (29 DEG)**	**20% O_2_ (197 DEG)**
membrane transport function genes down-regulated	cell growth and maintenance genes misregulated
signal transduction and cell adhesion genes upregulated	antioxidant response/possessing an antioxidant response gene upregulated
**Metabolites**	
**5% O_2_**	**20% O_2_**
Higher uptake of glucose and aspartate	Higher production of glutamate and ornithine

## Data Availability

Data is contained within the article.
